# Applications of the Natural Language Processing Tool ChatGPT in Clinical Practice: Comparative Study and Augmented Systematic Review

**DOI:** 10.2196/48933

**Published:** 2023-11-28

**Authors:** Nikolas Schopow, Georg Osterhoff, David Baur

**Affiliations:** 1 Department for Orthopedics, Trauma Surgery and Plastic Surgery University Hospital Leipzig Leipzig Germany

**Keywords:** natural language processing, clinical practice, systematic review, healthcare, health care, GPT-3, GPT-4, large language models, artificial intelligence, machine learning, clinical decision support systems, language model, NLP, ChatGPT, systematic, review methods, review methodology, text, unstructured, extract, extraction

## Abstract

**Background:**

This research integrates a comparative analysis of the performance of human researchers and OpenAI's ChatGPT in systematic review tasks and describes an assessment of the application of natural language processing (NLP) models in clinical practice through a review of 5 studies.

**Objective:**

This study aimed to evaluate the reliability between ChatGPT and human researchers in extracting key information from clinical articles, and to investigate the practical use of NLP in clinical settings as evidenced by selected studies.

**Methods:**

The study design comprised a systematic review of clinical articles executed independently by human researchers and ChatGPT. The level of agreement between and within raters for parameter extraction was assessed using the Fleiss and Cohen κ statistics.

**Results:**

The comparative analysis revealed a high degree of concordance between ChatGPT and human researchers for most parameters, with less agreement for study design, clinical task, and clinical implementation. The review identified 5 significant studies that demonstrated the diverse applications of NLP in clinical settings. These studies’ findings highlight the potential of NLP to improve clinical efficiency and patient outcomes in various contexts, from enhancing allergy detection and classification to improving quality metrics in psychotherapy treatments for veterans with posttraumatic stress disorder.

**Conclusions:**

Our findings underscore the potential of NLP models, including ChatGPT, in performing systematic reviews and other clinical tasks. Despite certain limitations, NLP models present a promising avenue for enhancing health care efficiency and accuracy. Future studies must focus on broadening the range of clinical applications and exploring the ethical considerations of implementing NLP applications in health care settings.

## Introduction

*The following manuscript was augmented by ChatGPT (versions 3.5 and 4.0; OpenAI [1]). ChatGPT-generated text is shown in Roman (unitalicized) font and has not been altered. Any modifications to the generated text, including corrections to sources or information, are explicitly indicated. Any text added or revised by human authors is shown in italics. All in-text reference citations have been reformatted to adhere to the journal’s style preferences.*

Natural Language Processing (NLP) has emerged as a powerful tool in recent years, enabling the processing and analysis of vast amounts of unstructured textual data in various domains, including healthcare and clinical practice [2] *(added [3])*. The application of NLP techniques in clinical settings has the potential to revolutionize the way medical professionals manage and analyze patient information, leading to improved patient outcomes, reduced costs, and increased efficiency in medical decision-making [4] *(added [5])*. 

In clinical practice, NLP can facilitate various tasks, such as disease diagnosis, treatment decision support, automation of clinical tasks, and data mining [6]. For instance, NLP algorithms have been used to screen and identify patients at risk for specific conditions [7], aid in the diagnosis of diseases by analyzing electronic health records (EHRs) [original: Demner-Fushman, D., & Chapman, W. W. (2017)] *(new [8])*, provide decision support in treatment planning [original: Wang, Y et al. (2017)] *(new [9])*, and automate routine clinical tasks [original: Devlin, J. et al. (2019)] *(new [10])*. Furthermore, NLP has been employed in the analysis of large-scale medical literature to identify trends, generate hypotheses, and inform clinical decision-making [original: Brown, T. B. et al. (2020)] *(new [11])*. 

Recent advancements in NLP, particularly the introduction of transformer-based models like Bidirectional Encoder Representations from Transformers (BERT) [original: Vaswani, A et al. (2017)] *(new [12])*, Generative Pre-trained Transformers (GPT) [original: Lee et al, 2020] *(new [13])*, and their variants *(moved [14])*, have significantly improved the performance of NLP tasks, including information extraction, question-answering, and text summarization. Transformer networks leverage attention mechanisms, allowing them to learn contextual relationships between words in a given text, thus enabling a more nuanced understanding of the input data [15].

Transformer-based models like BERT and GPT work using a self-attention mechanism, allowing them to focus on relevant words in a sentence, thus capturing contextual information efficiently [16]. This approach enables precise understanding of semantic relationships, making these models adept at tasks such as named entity recognition and text summarization [12].

However, these models have limitations. The attention mechanism is resource-intensive, potentially limiting their use in constrained environments [17]. Furthermore, while able to generate plausible-sounding outputs, they may occasionally produce nonsensical or incorrect results (called “artificial hallucination”), which emphasizes the need for careful interpretation [original: McCoy et al. 2019] *(new [18])*.

These models have been successfully applied to various healthcare-related tasks, including biomedical literature mining [19], clinical concept extraction [20] *(added [21])*, and predicting patient outcomes [original: Nye et al. 2018] *(new [22])*. 

Large language models (LLM) represent the cutting-edge of NLP, demonstrating exceptional performance in various tasks by leveraging their extensive pre-training on vast textual data [original: Smith et al., 2022] *(new [23])*.

Yet, despite the notable advancements in NLP and LLMs, traditional systematic reviews continue to pose significant limitations [24] *(added [25])*. Traditional approaches to systematic reviews are often labor-intensive and time-consuming, involving manual screening of literature and information extraction [26]. Such processes are not only susceptible to human error [27] but also struggle to cope with the exponential increase in available medical literature [28]. The extensive and complex nature of medical data, combined with the ever-evolving landscape of clinical research, presents a substantial challenge to traditional systematic review methods [29] *(added [30])*. Thus, there is a pressing need for more sophisticated and automated solutions, such as those provided by NLP, to handle the growing volume and complexity of medical literature [31].

In light of these developments, we aim to conduct a systematic review aided by NLP, specifically leveraging the capabilities of transformer-based models like GPT, to synthesize the existing literature on the application of NLP in clinical practice. Our review will focus on studies published between January 2020 and the present, evaluating the performance, implementation, and impact of NLP techniques in clinical settings. By integrating NLP into the systematic review process, we aim to increase the efficiency and accuracy of the review, enabling the identification of relevant studies, extraction of key information, and synthesis of findings in a more streamlined manner [32]. 

LLMs have been gaining traction in both social media and the scientific community. We compared the results of human researchers *(with a research experience of >7 years)* versus ChatGPT (Versions 3.5 and 4.0) in an artificial intelligence augmented systematic review. The goal was to explore the usefulness and limitations of LLMs in clinical practice, medical research and writing publications.

The main aim was to evaluate how effectively and reliably ChatGPT could support the process of conducting a medical systematic review, while also identifying potential issues and offering insights into the rapidly evolving field of artificial intelligence.

## Methods

### Overview

*The task of conducting a systematic review was augmented using ChatGPT. ChatGPT was used for general considerations in conducting a systematic review; determining MeSH (Medical Subject Headings) terms; title, abstract, and full-text screening; limited data extraction; and text generation.*

**Figure 1:**
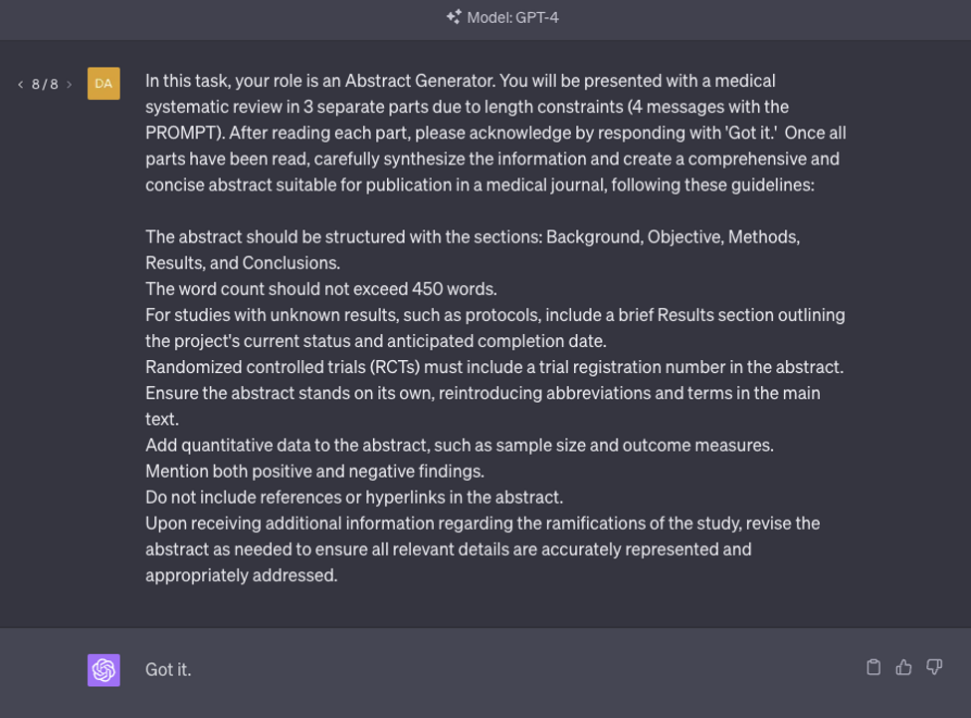
An exemplary prompt and response from ChatGPT as a multistep answer for prompt generation for the abstract text module.

*This manuscript was generated in several sections; therefore, modifications for better readability—for example, the order of text sections, numbering of references, and the use of abbreviations—are not shown. Relevant conversations with ChatGPT are provided in Figure 1 and Multimedia Appendices 1-16.*

**Figure 2:**
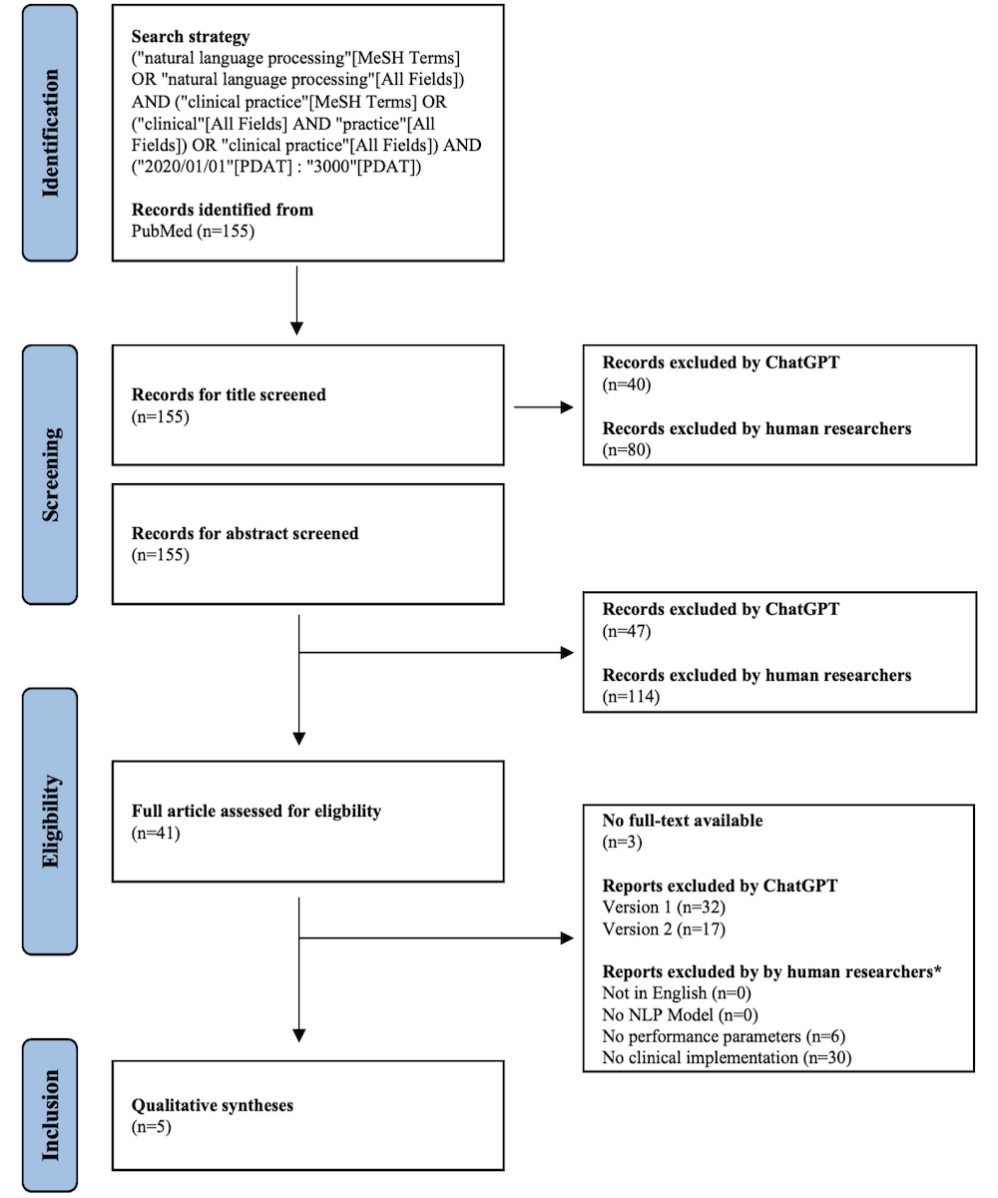
PRISMA (Preferred Reporting Items for Systematic Reviews and Meta-Analyses) flowchart. *In consensus, multiple reasons possible. MeSH: Medial Subject Headings; NLP: natural language processing.

Our systematic review followed the guidelines provided by the Cochrane Handbook for Systematic Reviews of Interventions [33] *(added [34])* and the PRISMA (Preferred Reporting Items for Systematic Reviews and Meta-Analyses) statement [25]. *The PRISMA flowchart is shown in Figure 2 and the PRISMA checklist in Multimedia Appendix 17.*

### Search Strategy 

We utilized ChatGPT 3.5 legacy (Version Jan 30, *OpenAI* *[1]*) to generate a MESH search strategy and define the inclusion and exclusion criteria for our review. *We repeated the prompts for MeSH term generation multiple times and refined the MeSH terms by narrowing overly broad terms, incorporating essential terms that were initially omitted by ChatGPT and excluding terms that were not relevant to our review.* Two human researchers, NS and DB, used the MESH terms generated for the PubMed search and retrieved a total of 155 articles. They prepared the articles for presentation to ChatGPT, presenting only the title for title screening, only the abstract for abstract screening, and only the text of the Introduction, Methods, Results, and Discussion for full-text screening, *without any reference to authors or publishing journal, etc*. NS and DB also created all the prompts for ChatGPT and saved all interactions with the Transformer network. These interactions will be made available as supplementary materials. 

### Screening Process 

Title and abstract screening were conducted independently by ChatGPT 3.5 legacy and the two human researchers, NS and DB. Abstracts were only included for full-text analysis when a consensus was reached between ChatGPT and the human researchers (n=41). NS and DB then generated a table for structured data extraction at the full-text screening level, which will be included in the paper.

Two separate instances of ChatGPT 3.5 legacy were used to independently screen all full texts prepared by NS and DB. NS and DB also evaluated all full-text articles (n=41) for inclusion or exclusion.

### Data Extraction and Synthesis 

The review of the five included articles was conducted by ChatGPT 4.0 (Version March 15). First, ChatGPT summarized each paper. Next, it was asked to generate a results section and discussion section. All authors extracted data from the included papers and reviewed the text generated by ChatGPT 4.0, making any necessary adjustments and adaptations. *Additionally, tables and charts were generated by human researchers, owing to the constraints of ChatGPT at the time of conducting this study. We extracted the following items in the extraction table (Table S1 in Multimedia Appendix 18): English language (yes/no), targeted disease, study design (randomized controlled trial; cohort study; cross-sectional study; case report or series; meta-analysis, systematic review, or review; opinion; others, experimental, or not applicable), NLP model (yes/no), sample size, performance parameters available (yes/no), clinical task (screening or risk, disease diagnosis, treatment decision, decision support, automation of clinical tasks, data mining or automated document evaluation, others, or not applicable), and clinical implementation (yes/no). The reference directory was compiled by human researchers.*

### Statistical Analysis of GPT and Human Performance

*In this study, we used several standard performance metrics to evaluate the effectiveness of the search strategy generated by ChatGPT. Below, we describe the calculation of each of these metrics.*

Sensitivity (also known as True Positive Rate): Sensitivity is calculated as the number of true positives (TP) divided by the sum of the true positives and the false negatives (FN).

Sensitivity=TP(TP+FN)

Specificity: Specificity is calculated as the number of true negatives (TN) divided by the sum of the true negatives and the false positives (FP).

Specificity=TN(TN+FP)

Precision (also known as Positive Predictive Value): Precision is calculated as the number of true positives (TP) divided by the sum of the true positives and the false positives (FP).

Precision=TP(TP+FP)

Accuracy: Accuracy is calculated as the sum of the true positives (TP) and true negatives (TN) divided by the sum of the true positives, true negatives, false positives, and false negatives.

Accuracy=(TP+TN)(TP+TN+FP+FN)

Chance Hit Rate: The Chance Hit Rate is calculated as the sum of the product of sensitivity and prevalence, and the product of specificity and (1-prevalence).

Chance Hit Rate=(Sensitivity⋅Prevalence)+(Specificity⋅(1−Prevalence))

*Statistical analysis was carried out by the human researchers due to limitations of ChatGPT at that time. The inter- and intrarater reliability, sensitivity, specificity, and other statistics were calculated using the inclusion/exclusion table created by all authors. The extracted data were compared using Fleiss and Cohen κ, correlating results from both human researchers and 2 iterations of ChatGPT 3.5 [35,36]. Consensus among human researchers was considered the gold standard to compare the performance of the human researchers with that of ChatGPT 3.5 iterations. When a specific measure was not considered as an item, we used a binary categorization of “correct” or “incorrect” as the items (ie, sample size) for assessing inter- and intrarater reliability, decided by consensus with consultation of a third human researcher.*

## Results

### MESH Search and Screening Process

Our MESH search on PubMed yielded 155 papers. Upon screening the titles, the two human researchers included 75 papers, while ChatGPT 3.5 included 115, achieving a sensitivity of [original: 97.33%] *100%* and specificity of [original: 37.5%] *50%* *(precision=65.2%, accuracy=74.2%, and chance hit rate=49.2%)*. Following the abstract screening of all 155 abstracts, the two human researchers included 41 articles, while ChatGPT 3.5 included 108, resulting in a sensitivity of [original: 95.12] *100%* and specificity of [original: 34.38] 41.2% *(precision=39.6%, accuracy=56.8%, and chance hit rate=40.7%)*. A total of [original: 38] *41* articles were selected for full-text analysis, with 3 articles being excluded due to unavailability. Ultimately, 5 articles were incorporated into our systematic review [37–41].

### Natural Language Processing Applications in Various Clinical Settings

#### Clinical Decision Support System (CDSS) for Concept-Based Searching

Berge et al. developed a machine learning-driven CDSS employing NLP for concept-based searching in a Norwegian hospital [37]. The study introduced an Information System for Clinical Concept-based Search (ICCS) CDSS, devised to detect patient allergies in EHRs using unsupervised machine learning algorithms for clinical narrative analysis. The system combines unsupervised and supervised algorithms with deterministic rules to enhance precision. In a previous study, the ICCS achieved a recall of 92.6%, precision of 88.8%, and F-measure of 90.7%. The ICCS aims to improve allergy detection and classification, thereby enhancing patient safety in anesthesia and ICU settings.

#### Digital Pathology Applications

Marchesin et al. investigated the use of NLP to strengthen digital pathology applications [38]. The authors introduced explainable knowledge extraction tools capable of extracting pertinent information from pathology reports. They presented the Semantic Knowledge Extractor Tool (SKET), a hybrid knowledge extraction system for digital pathology applications. SKET combines expert knowledge, pre-trained machine learning models, and rule-based techniques such as ScispaCy. The tool exhibits high performance in entity linking and text classification tasks across various cancer use-cases, surpassing unsupervised approaches. The web-based system, SKET X, enables domain experts to understand SKET's outcomes, rules, and parameters for explainable AI. Applications include automatic report annotation, pathological knowledge visualization, and Whole Slide Image classification.

#### Identification of Nonvalvular Atrial Fibrillation (NVAF)

Elkin et al. employed artificial intelligence with NLP to integrate electronic health record (EHR) structured and free-text data to identify NVAF, aiming to reduce strokes and death [39]. The study utilized high-definition NLP (HD-NLP) to process free text in EHRs, identifying patients with Nonvalvular Atrial Fibrillation (NVAF) and estimating their stroke and bleeding risks. NLP-assisted analysis of structured and unstructured EHR data improved detection rates and accuracy compared to structured data alone. This approach could potentially prevent 176,537 strokes, 10,575 deaths, and save over $18 billion in the first year if implemented nationally, with a net financial benefit of approximately $14.4 billion.

#### Cardiovascular Disease Comorbidity Assessment

Berman et al. applied NLP to assess cardiovascular disease comorbidities in the Cardio-Canary Comorbidity Project [40]. The authors demonstrated the potential of NLP in facilitating the identification of comorbidities, leading to improved patient care and outcomes in cardiovascular disease management. The modules exhibited robust performance, particularly for hypertension, dyslipidemia, and stroke, with over 95% positive predictive value (PPV) for note-level performance. The NLP modules provide an accurate, open-source system for various applications, such as population management, clinical research, and clinical trial recruitment.

#### Post-traumatic stress disorder (PTSD) Quality Metrics Improvement

Shiner et al. explored the use of NLP to enhance PTSD quality metrics in psychotherapy treatments for veterans [41]. The study combined structured EMR data with NLP-derived data to evaluate PTSD care quality in the Veteran Affairs system. The validated NLP algorithm displayed a high degree of agreement with template data (weighted kappa: 0.81), capturing nearly 90% of evidence based psychotherapy for PTSD visit days. The study revealed that 20% of PTSD checklist values were documented exclusively in free-text clinical notes. The findings suggest that NLP can bridge documentation gaps, provide a more comprehensive view of care quality, and improve measurement practices for PTSD patients within the Veterans Affairs healthcare system.

### Comparison Between ChatGPT and Human Researchers

*Except for clinical tasks (*κ*=0.56), both human researchers showed very good agreement (*κ*>0.90) for the parameters extracted from the included articles (Table 1). ChatGPT and the human researchers showed very good agreement for the article’s language (*κ*=1), targeted disease (*κ*=1), NLP model (*κ*=0.95), sample size (*κ*=0.83), and performance parameters (*κ*=0.85); good agreement for study design (*κ*=0.79); moderate agreement for clinical task (*κ*=0.58); and only fair agreement for clinical implementation (*κ*=0.34). All numbers were extracted correctly from the articles by ChatGPT.*

*In the process of composition, ChatGPT was prompted to provide source citations (refer to Table S2 in Multimedia Appendix 19). Among the 28 references supplied, 3 were found to be fictitious: Smith, Brown & Lee (2022), Demner-Fushman & Chapman (2017), and McCoy, Hughes, Jao & Perlis (2019); this rendered the attribution of Smith, Lee and Joa uncertain. Although the other authors have multiple publications within the NLP domain, a reliable attribution remains elusive. Five of the references were thematically pertinent, yet they did not accurately substantiate the statements made. Additionally, 2 sources required corrections to their publication years. Consequently, a total of 15 references were amended, appended, or substituted.*

**Table 1:** Inter- and intrarater reliability for extraction items using Fleiss and Cohen κ.

	All (Fleiss κ)	GPT^a^ 3.5 1 vs 2 (Cohen κ)	Human researcher 1 vs 2 (Cohen κ)
Language	1	1	1
Targeted disease	1	1	1
Study design	0.7333676	0.78939034	0.94736842
NLP^b^ model	0.1441441	0.947331947	1
Sample size	–0.041096	0.829723674	0.91486184
Performance	0.6847407	0.853733641	0.9425548
Clinical Task	0.5615047	0.58372457	0.56422018
Implementation	0.3531915	0.34127844	0.92132505

^a^GPT: Generative Pre-Trained Transformer.

^b^NLP: natural language processing.

## Discussion

This systematic review aimed to investigate the current natural language processing (NLP) models being used in daily clinical practice. We identified five studies that showcased various applications of NLP in clinical settings, including clinical decision support systems, digital pathology applications, identification of nonvalvular atrial fibrillation, cardiovascular disease comorbidity assessment, and PTSD quality metrics improvement. These studies highlight the potential of NLP to revolutionize healthcare by improving efficiency, accuracy, and patient care.

Berge et al. [37] presented a clinical decision support system (CDSS) that uses NLP for concept-based searching in a Norwegian hospital. Their study demonstrated the potential of machine learning-driven CDSS to improve allergy detection and classification, leading to enhanced patient safety in anesthesia and ICU settings. Marchesin et al. [38] focused on the application of NLP in digital pathology applications, showcasing how NLP can support pathologists and improve the overall quality of pathology diagnosis and patient care. Elkin et al. [39] showed the effectiveness of NLP in identifying NVAF patients, which has the potential to lead to better management of NVAF and prevent strokes and death. Berman et al. [40] utilized NLP for cardiovascular disease comorbidity assessment, illustrating the potential of NLP to facilitate the identification of comorbidities, leading to improved patient care and outcomes. Lastly, Shiner et al. [41] examined the use of NLP to improve PTSD quality metrics in psychotherapy treatments for veterans, demonstrating NLP's value in capturing important data in large healthcare systems and improving measurement practices.

The studies included in this review showcased various NLP techniques, such as machine learning algorithms, rule-based techniques, and the use of pre-trained models like ScispaCy. These approaches demonstrate the versatility of NLP in handling different clinical tasks and highlight the potential for continued development in this field. Moreover, the use of transformer-based models like GPT-3 in conducting this systematic review serves as an example of how NLP can improve the efficiency and accuracy of literature synthesis in a streamlined manner [32].

Despite the promising results, the studies included in this review also have some limitations. First, the studies are limited in terms of the variety of clinical applications and settings, as only five studies were included in the review. This could potentially limit the generalizability of the findings. Furthermore, the studies may have inherent biases and limitations that could impact the interpretation of the results. It is essential to be cautious when extrapolating these findings to other contexts and clinical settings.

Future research should focus on expanding the range of clinical applications and settings where NLP can be utilized, as well as investigating the scalability and generalizability of the identified approaches. Additionally, more studies should be conducted to explore the potential of transformer-based models like GPT-3 and BERT in clinical practice. These models have shown great promise in various NLP tasks and may offer further advancements in the field of healthcare.

In conclusion, our systematic review highlights the potential of NLP in revolutionizing clinical practice by improving efficiency, accuracy, and patient care. The studies included in this review showcase various NLP applications in clinical settings, demonstrating the versatility and potential for growth in this field. Further research is needed to expand the range of clinical applications and settings, as well as to explore the potential of transformer-based models in healthcare. As NLP continues to advance, it is expected that its impact on clinical practice will only increase, leading to improved patient outcomes and more efficient healthcare systems. 

## Concluding Remarks by the Human Authors

*Concerning the systematic review, we only searched PubMed and no other database or registry. Furthermore, the MeSH search generated only 155 hits, and we must admit that this study does not allow us to determine whether NLPs are of practical use in clinical practice today. Since the MeSH term itself was produced by ChatGPT and the main goal of this study was to explore the usefulness of ChatGPT in performing or assisting in systematic reviews, we adhered to the generated methods; however, this compromises the quality of the systematic review. Therefore, we do not believe that an adequate commentary on the state and usefulness of NLP in clinical practice is within the scope of this study. During the research, ChatGPT underwent several updates. We attempted to split workflows for both GPT 3.5 and GPT 4.0. Since developments on LLMs change at a fast pace, their applicability might change fast as well, which means that results from interactions and the idea of augmented or automated systematic reviews can change drastically, for example, with developments in LLMs’ ability to access of databases such as PubMed or Cochrane.*

*We hypothesize that automated systematic reviews could become a reality in the near future. However, the current state of ChatGPT versions 3.5 and 4.0, with their multiple limitations, renders augmented systematic reviews inefficient for experienced researchers. Yet, for language correction, particularly for nonnative English speakers, and rectification of grammatical errors, or for text condensation and modification in form and wording, it proves to be of significant value. As we confined our study to ChatGPT, without the use of any plugins or application programming interface implementations, we anticipate that the forthcoming months or years will witness an increased application of LLMs in scientific research, showcasing intriguing architectures such as “Lang Chain” and “Agent GPT” as pioneering examples of more complex programs powered by LLMs.*

*Ethical and legal concerns about the implementation of LLMs in a scientific field as sensible as medicine have led to an ongoing discussion and should be considered before broadening the spectrum of clinical applications for NLP-driven automations. The black box issue associated with LLMs such as ChatGPT, even when using the most deterministic options, is an undeniable fact. Automatic analyses of all available literature within minutes or seconds, however, would change the way we conduct research or are able to access information in clinical practice. Further research is imperative, accompanied by a debate on the ethical implications of such potent tools and strategies to oversee and regulate the use of these models in scientific writing.*

## Appendix

Multimedia Appendix 1 Article sumarization.

Multimedia Appendix 2 Article sumarization.

Multimedia Appendix 3 Data extraction.

Multimedia Appendix 4 Extraction fulltext.

Multimedia Appendix 5 Full text analysis GPT1.

Multimedia Appendix 6 Full text analysis GPT2.

Multimedia Appendix 7 MESH-Term inclusion/exclusion criteria.

Multimedia Appendix 8 Title screening GPT1.

Multimedia Appendix 9 Title screening GPT2.

Multimedia Appendix 10 Abstract screening GPT1.

Multimedia Appendix 11 Writing abstract and title.

Multimedia Appendix 12 Writing methods.

Multimedia Appendix 13 Writing results and conclusion.

Multimedia Appendix 14 Rewrite results.

Multimedia Appendix 15 Supplementary Materials, Task and Prompts correlated.

Multimedia Appendix 16 Supplementary Materials, Task and Prompts correlated.

Multimedia Appendix 17 PRISMA 2020 Checklist.

Multimedia Appendix 18 Two human researchers (NS, DB) and two instances of ChatGPT (GPT1, GPT2) were tasked with individually checking the full text of the articles for inclusion and exclusion criteria, as well as extracting data endpoints.

Multimedia Appendix 19 Control of the references.

## Abbreviations

BERT: Bidirectional Encoder Representations from Transformers
CDSS: clinical decision support system
EHR: electronic health record
FN: false negative
FP: false positive
GPT: Generative Pre-trained Transformer
ICCS: Information System for Clinical Concept-based Search
LLM: large language model
MeSH: Medial Subject Headings
NLP: natural language processing
NVAF: nonvalvular atrial fibrillation
PRISMA: Preferred Reporting Items for Systematic Reviews and Meta-Analyses
PTSD: posttraumatic stress disorder
SKET: Semantic Knowledge Extractor Tool
TN: true negative
TP: true positive
